# Dynamic changes in water and salinity in saline-alkali soils after simulated irrigation and leaching

**DOI:** 10.1371/journal.pone.0187536

**Published:** 2017-11-01

**Authors:** Shutao Wang, Qian Feng, Yapeng Zhou, Xiaoxi Mao, Yaheng Chen, Hao Xu

**Affiliations:** 1 Land and Resources College, Hebei Agricultural University, Baoding, China; 2 Key Laboratory of Hebei Agricultural Environment, Baoding, China; 3 Cangzhou Field Research Station, Land Use of Circum Bohai Sea for the Ministry of Land and Resources, Baoding, China; 4 Plant Protection Institute, Hebei Academy of Agriculture and Forestry Sciences, Baoding, China; Institute of Genetics and Developmental Biology Chinese Academy of Sciences, CHINA

## Abstract

Soil salinization is a global problem that limits agricultural development and impacts human life. This study aimed to understand the dynamic changes in water and salinity in saline-alkali soil based on an indoor soil column simulation. We studied the changes in the water and salt contents of soils with different degrees of salinization under various irrigation conditions. The results showed that after seven irrigations, the pH, conductivity and total soluble salt content of the percolation samples after irrigation generally increased initially then decreased with repeated irrigation. The soil moisture did not change significantly after irrigation. The pH, conductivity, and total soluble salt content of each layer of the soil profile exhibited general declining trends. In the soil profile from Changguo Township (CG), the pH decreased from 8.21–8.35 to 7.71–7.88, the conductivity decreased from 0.95–1.14 ms/cm to 0.45–0.68 ms/cm, and the total soluble salt content decreased from 2.63–2.81 g/kg to 2.28–2.51 g/kg. In the soil profile from Zhongjie Industrial Park (ZJ), the pH decreased from 8.36–8.54 to 7.73–7.96, the conductivity decreased from 1.58–1.68 ms/cm to 1.45–1.54 ms/cm, and the total soluble salt decreased from 2.81–4.03 g/kg to 2.56–3.28 g/kg. The transported salt ions were primarily K^+^, Na^+^ and Cl^-^. After several irrigations, a representative desalination effect was achieved. The results of this study can provide technical guidance for the comprehensive management of saline-alkali soils.

## Introduction

Soil salinization is a global problem that limits agricultural development and impacts human life. Saline-alkali soil results from continuous increases in the salt and alkali metal contents in the soil or soil components. This process can cause non-salinized soil to transform into salinized soil under natural or artificial conditions. The transport of soil water and salt is a temporally and spatially dynamic process [1–3], and studying this process can provide a theoretical foundation for the development and utilization of saline-alkali soil in coastal zones.

Many scholars have studied soil water and salt transport. Soil water plays a dominant and decisive role in the transport of water and salt [4–6], during which soil is the carrier and salt is a soil component that is closely related to the soil moisture movement. Therefore, the transport of soil moisture and that of salt are intertwined, and the dynamic changes in these two components can be used to evaluate the transport processes and associated trends [7–10]. Siyal et al. studied the transport of soil solutes under the effects of subsurface irrigation using a porous clay pipe [11]. Phogat et al. used the HYDRUS-2D model to simulate and analyze the soil water and salt dynamics under continuous drip irrigation conditions [12]. Benduhn and Renard proposed a dynamic model of water and salt balance in saline-alkali areas [13]. Musa et al. [14] and Hui et al. [15] studied the mechanisms of soil water and salt migration during soil freeze-thaw cycles. Selim et al. analyzed the soil water and salinity distributions under surface drip irrigation [16]. Hu et al. established a model of soil water and salt transport in inland arid areas and studied the soil water and salt transport trends in these areas [17]. Li et al. compared and analyzed the temporal variations in soil water and salt in a saline-alkali soil in an arid region under different irrigation quotas [18]. However, most of these studies concentrated on arid inland areas, and few comprehensive calibration analyses of the soil water and salt transport parameters have been performed.

Huanghua, located in the eastern coastal area of China, contains abundant undeveloped land. However, most of its soils are saline-alkali; these soils are high in salinity and of poor quality, resulting in low land use efficiency. Overall, 14.1% of the total land area of the city, i.e., an area of 33,715.33 hm^2^, is a saline wasteland containing Cl-SO_4_^2—^Na salinized soil, and the total salt content in the topsoil ranges from 2.4% to 18.5%, reflecting moderate to severe saline-alkali soil [19]. Gao studied the soil water and salt dynamics and the saline-alkali soil improvements resulting from the presence of salt-tolerant plants in the saline-alkali soils of Bohai Bay (including Huanghua) [20]. Wang studied the salinity of a saline-alkali soil and the variations in salt ions and nutrients in Huanghua [21]. However, the dynamic changes in water and salt transport have not been studied.

To address these soil salinization problems, the soil water and salt transport trends in Huanghua were simulated. Based on a summary of the soil salinization degree and hydrogeological data from Huanghua, we conducted simulation experiments involving soil column irrigation to determine the parameters of soil moisture transport and studied the dynamic changes in soil moisture and salt during irrigations. A better understanding of the dynamic changes in water and salt in saline-alkali soils can provide a theoretical basis for improving agricultural development in areas with similar geographical conditions around the world.

## Materials and methods

### General conditions in the study area

Huanghua is located in the “key area” of the Bohai Sea and the Beijing-Tianjin region at 117.08°-117.82°E and 38.15°-38.65°N. The city has a warm temperate semi-humid monsoon climate and certain maritime climate characteristics due to its proximity to the Bohai Sea. The climate alternates between cold and warm weather conditions, as well as between dry and wet conditions. The area is prone to droughts, floods, storm tides, red tides and other weather-related disasters. Huanghua has an average annual temperature of approximately 12°C and an average sunshine duration of approximately 2,755 h. The annual average evaporation is greater than 1,908 mm, and the average precipitation is 574 mm. The soil of Huanghua consists of three types (fluvo-aquic soil, saline-alkali soil and marsh soil) and seven subtypes. The fluvo-aquic soil type includes three subtypes: coastal fluvo-aquic soil, coastal salinized fluvo-aquic soil and coastal swamp fluvo-aquic soil. The coastal areas of Huanghua contain saline-alkali soils.

### Experimental design

#### Experimental methods

Based on indoor analysis data from the topsoil and soil profiles sampled in April 2015, representative soils from Changguo Township (CG) and Zhongjie Industrial Park (ZJ) with salt contents of 2.6 g/kg and 4.3 g/kg, respectively, were selected for further analysis (Table 1). The experimental setup consisted of a water supply device connected to the soil column and a water collection device at the bottom. The soil samples of each profile were sorted and then placed in the soil column in preparation for the indoor soil column test. The local crops in the investigated regions are mainly wheat and maize, whose roots mainly grow in the 0–20 cm layer of soil. The topsoil is light or medium loam, with a physical clay content between 25.06% and 44.46%. The local corn growth period extends from May to September, and irrigation is concentrated between April and August. The irrigation water quota is 4875 m^3^/hm^2^, which is equivalent to a water layer thickness of 487.26 mm. Additionally, precipitation is considered a soil water source from April to September, when an average of 310.4 mm of precipitation is received by the area. Therefore, the water supplied to the local soil from April to September is as follows: 487.26 mm + 310.4 mm = 797.66 mm = 79.77 cm. The inner diameter of the test soil column was 15.0 cm, the cross-sectional area was 176.62 cm^2^, and the thickness of the irrigation water layer was 79.77 cm. Therefore, the amount of water required is as follows: V = 176.62×79.77 = 14,088.97 cm^3^ = 14,088.97 mL. This approximately 14,000 mL was divided into seven irrigation applications of 2,000 mL each. After each irrigation event, the soil percolation was measured, and a soil sample from each layer was collected to measure the water and salt contents. These values were compared with those before irrigation to analyze the irrigation-induced trends in the soil water and salt contents. The experiment included three parallel samples for each treatment.

**Table 1:** Distribution of salt ions in the soil profile.

Soil sample	Depth of soil layer (cm)	K^+^ (g/kg)	Na^+^ (g/kg)	HCO^3-^ (g/kg)	Cl^-^ (g/kg)	Ca^2+^ (g/kg)	Mg^2+^ (g/kg)	SO_4_^-^ (g/kg)	Total amount of soluble salt (g/kg)	Conductivity (ms/cm)
CG										
	0–20	0.16	0.28	0.97	0.27	0.45	0.22	0.29	2.65	1.07
	20–40	0.10	0.23	0.92	0.29	0.54	0.30	0.26	2.63	1.06
	40–60	0.14	0.36	1.02	0.34	0.41	0.27	0.23	2.76	1.14
	60–80	0.19	0.36	0.94	0.41	0.36	0.29	0.26	2.81	1.11
	80–100	0.16	0.30	0.79	0.44	0.44	0.29	0.24	2.67	0.95
ZJ										
	0–20	0.26	0.78	0.68	1.25	0.43	0.27	0.36	4.03	1.68
	20–40	0.19	0.64	0.44	1.28	0.41	0.11	0.44	3.53	1.68
	40–60	0.16	0.44	0.63	1.14	0.38	0.13	0.23	3.11	1.60
	60–80	0.21	0.37	0.37	0.85	0.52	0.16	0.33	2.81	1.63
	80–100	0.25	0.46	0.52	1.03	0.56	0.49	0.38	3.70	1.58

All soil samples in this study originated from deserted saline-alkali land. Therefore, no specific permissions were required for the sampling process. Furthermore, this study did not involve any endangered or protected species. The water samples for simulating irrigation were taken from local wells, and the sampling process was approved by the local farmers.

#### Experimental procedures

**Fig 1:**
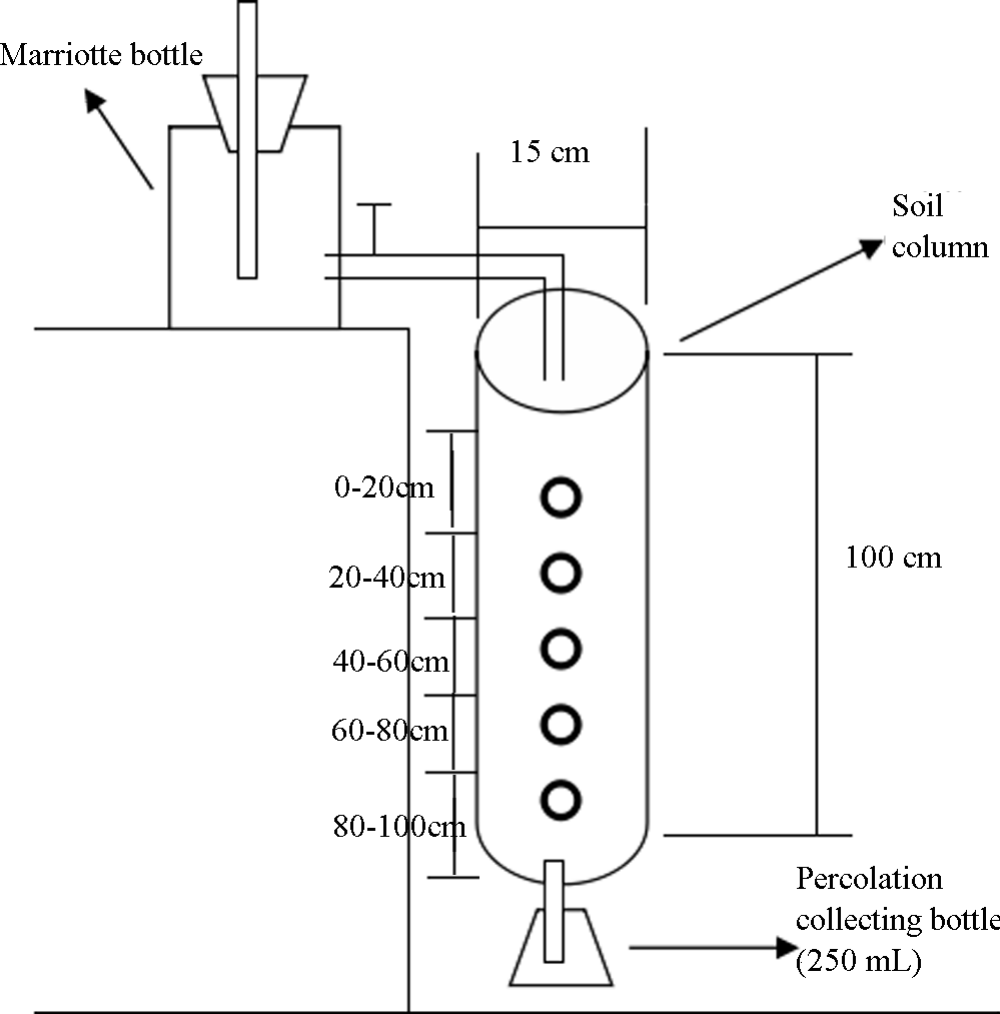
Diagram of the indoor soil column setup.

The soil column consisted of a PVC tube with an inner diameter of 15 cm, a length of 100 cm, and a supporting PVC base. Holes were drilled in the middle of the base to permit soil percolation, as shown in Fig 1. Before filling the column with soil, a filter net was placed at the bottom of the soil column. Then, a 2–3 cm layer of quartz sand was used as the filter layer. Next, the previously prepared soil was placed in the soil column according to the original soil profile. Finally, the top of the soil column was covered with a filter net and a 2–3 cm-thick layer of quartz sand to protect the surface soil from the effects of water addition. The soil column was saturated using a Mariotte bottle. The water siphon of the Mariotte bottle was connected to the soil column at the water receiving position. Saturation from the top layer to the bottom layer was achieved using the gradient permeation method with deionized water. When water reached the bottom of the soil column, saturation was complete. After the soil column was saturated, local well water with a total soluble salt content of 116.00 mg/L was used for irrigation. Irrigation with 2,000 mL of the well water was performed once every four days. Percolation occurred in the soil column during each irrigation event, and a small earth-boring auger was used to sample each soil layer before the next irrigation event. Then, various indexes were calculated. The irrigation amount was determined according to the local precipitation, irrigation and evaporation.

Soil sampling was performed when percolation ceased after irrigation. The soil was removed from the column, placed in an aluminum specimen box, and weighed immediately. The soil moisture content was determined using the oven drying method. The remaining soil sample was naturally air dried, ground, and sieved (1 mm). The soil was extracted with water at a ratio of 1 to 2.5, and the soil extract was analyzed to determine the pH, conductivity, and the total and individual contents of soluble salt ions in the soil. Then, the obtained data were analyzed.

## Results and discussion

### Change in percolation after irrigation

**Fig 2:**
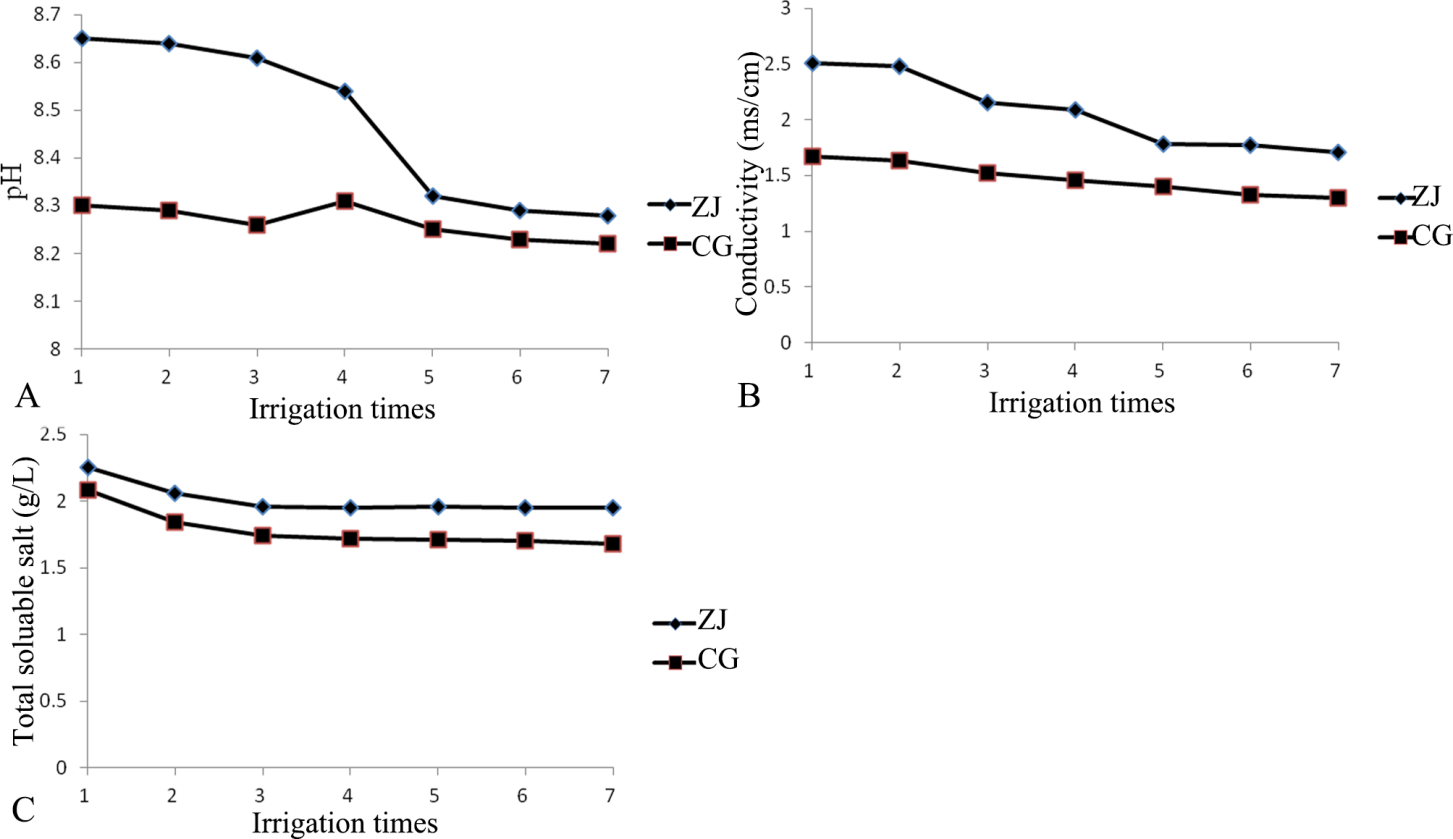
Changes in the pH, conductivity and total soluble salt in the percolation water samples from CG and ZJ.

According to Fig 2, the pH of the percolation water after the first irrigation was 8.65 in the ZJ profile. As the number of irrigation applications increased, the pH gradually decreased. After the seventh irrigation, the pH had decreased to 8.28. The conductivity and total soluble salt content after the first irrigation were 2.51 ms/cm and 2.25 g/L, respectively. As the number of irrigation applications increased, these values gradually decreased to 1.71 ms/cm and 1.95 g/L, respectively.

The pH of the percolation sample after the first irrigation was 8.3 in the CG profile. As the number of irrigation applications increased, the pH gradually decreased. After the seventh irrigation, the pH had decreased to 8.22. The conductivity and total soluble salt content after the first irrigation were 1.67 ms/cm and 2.08 g/L, respectively, and these values decreased to 1.33 ms/cm and 1.68 g/L, respectively, as the number of irrigation applications increased.

Comparing these two profiles, the pH, conductivity, and total soluble salt content of the soil sample from the ZJ profile were higher than those from the CG profile. Therefore, under the same irrigation conditions, these parameters were higher in the ZJ percolation sample than in the CG percolation sample due to desalination effects.

After the first irrigation, the pH, conductivity and total soluble salt content of the percolation samples were higher than the initial conditions. However, as irrigation progressed, these parameters gradually decreased. Similarly, Yang found that the pH, conductivity and total soluble salt content of percolation samples initially increased then decreased after irrigation [22]. These results suggested that after several irrigations, the soil water in the soil column flowed out of the column due to the irrigation water flow and removed ions that had leached from the soil into the percolation water. As the number of irrigation applications increased, the salt content in the profile decreased; therefore, the salt content in the percolation samples also decreased. Therefore, in this case, irrigation led to salt leaching in the profile.

### Soil moisture changes after irrigation

**Fig 3:**
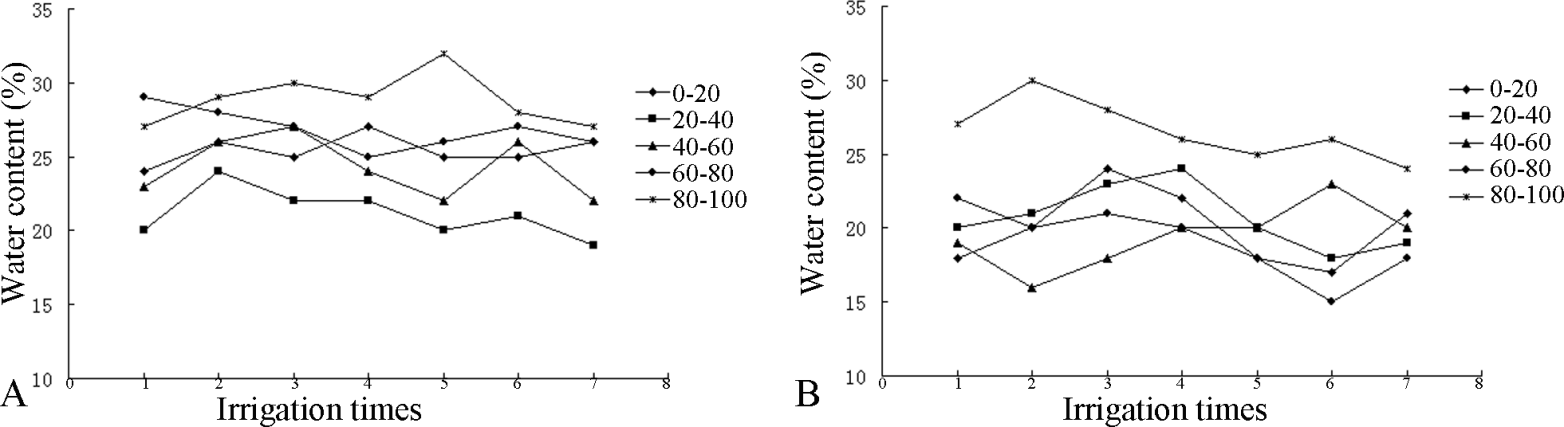
Changes in the soil water content in the CG and ZJ profiles.

According to Fig 3, the water content was highest in the 60–100 cm layer and lowest in the 20–40 cm layer of the CG soil column. Additionally, the soil water contents after the second, fourth and sixth irrigations were all higher than those after the other irrigations. In the ZJ profile, the water contents in the 20–40 cm and 80–100 cm layers were relatively high, and the water contents in the 0–40 cm and 80–100 cm layers after the fourth irrigation were similar, which were lower than those in the other two layers.

The water content in each layer of the soil profile did not change significantly, but the change in the water content in each layer varied after each irrigation, exhibiting both increases and decreases. The water content in the CG profile varied from 19%-32%, while that in the ZJ profile varied from 15%–30%. A related study showed that the water content in each layer of a soil profile fluctuated after each irrigation [22], which is consistent with the results of this study.

### Soil salinity changes after irrigation

**Fig 4:**
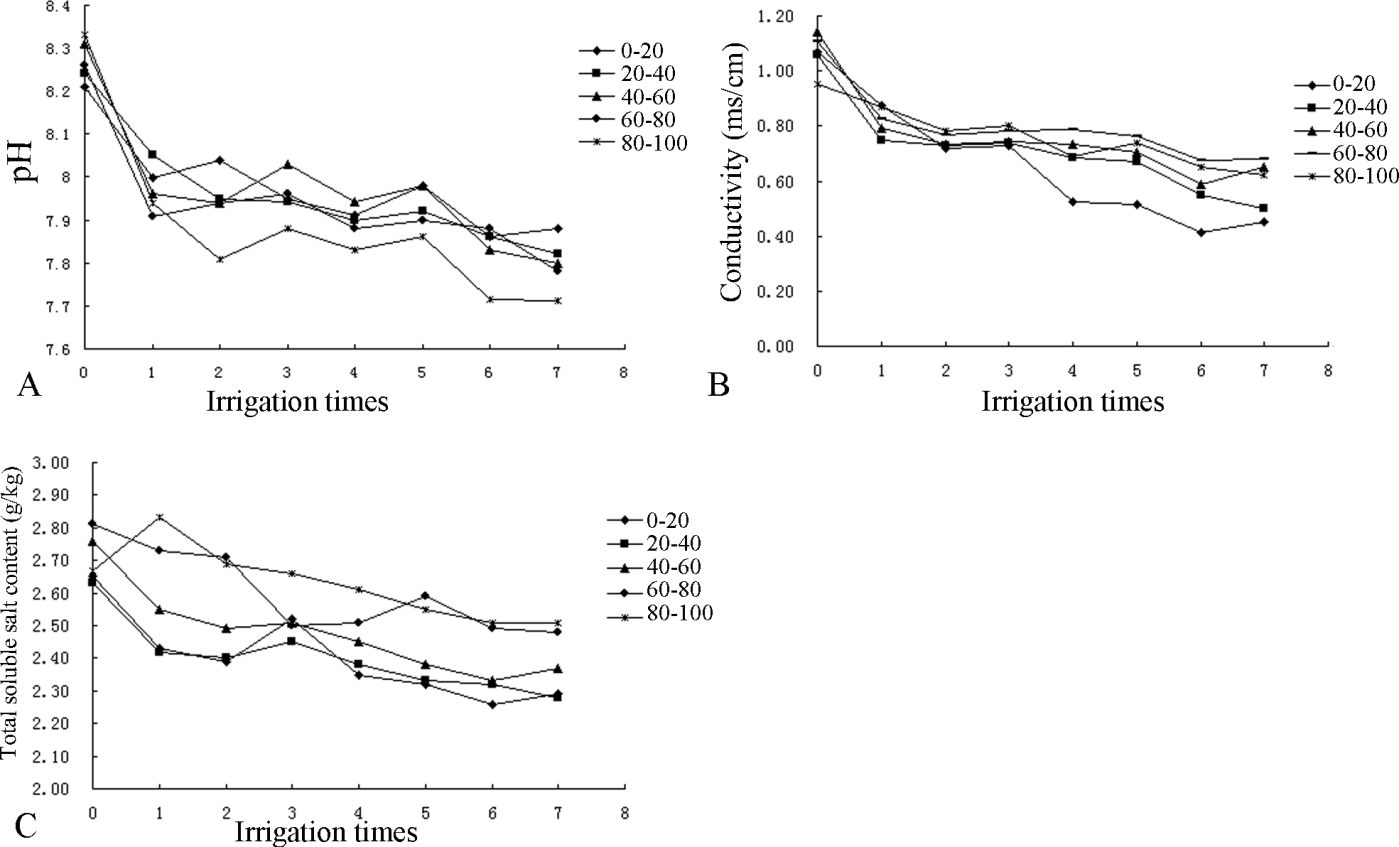
Changes in the pH, conductivity and total soluble salt content of the CG profile.

**Fig 5:**
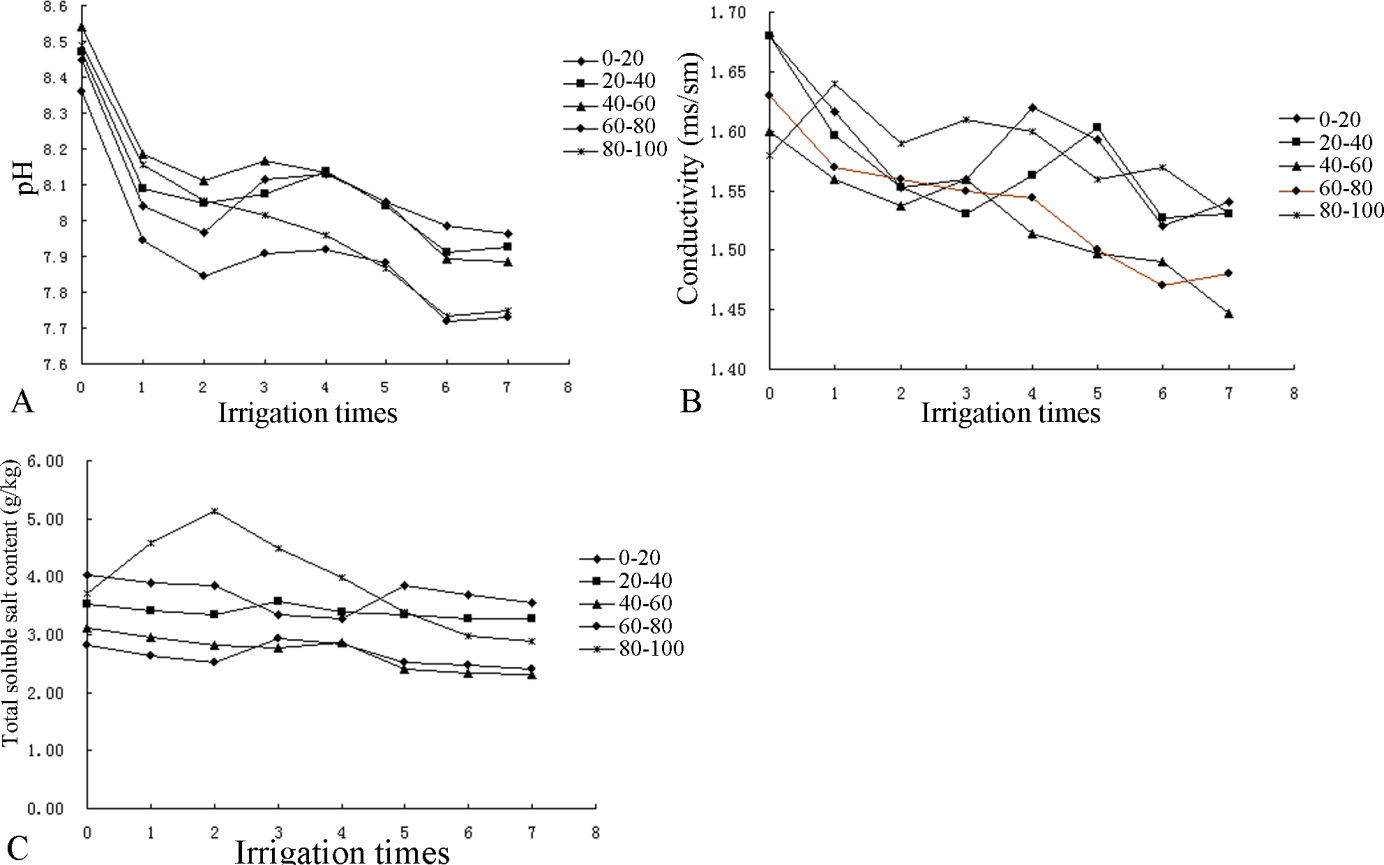
Changes in the pH, conductivity and total soluble salt of the ZJ profile.

According to Figs 4 and 5, after seven irrigations, the pH, conductivity, and total soluble salt content of each layer of the soil profile had decreased. (The soluble salt ion contents in the soil and the conductivity values after irrigation are summarized in Table 2.) Although some increases and decreases in the parameters after each irrigation, the values of all parameters were smaller than those before irrigation. Overall, the pH and salt content of the overall profile decreased, indicating that irrigation had a leaching effect on the salt in the profile.

**Table 2:** Soluble salt ion contents and the conductivity of the soil after irrigation.

Soil sample	Depth of soil layer (cm)	K^+^ (g/kg)	Na^+^ (g/kg)	HCO^3-^ (g/kg)	Cl^-^ (g/kg)	Ca^2+^ (g/kg)	Mg^2+^ (g/kg)	SO_4_^-^ (g/kg)	Total amount of soluble salt (g/kg)	Conductivity (ms/cm)
CG										
	0–20	0.03	0.15	0.53	0.15	0.26	0.21**	0.21**	1.54**	0.45*
	20–40	0.03*	0.11	0.53	0.15	0.22**	0.25	0.15*	1.44**	0.50
	40–60	0.04	0.22*	0.59*	0.21	0.17	0.20	0.11	1.54	0.65**
	60–80	0.06	0.20**	0.53	0.22	0.51	0.18	0.13	1.83**	0.68
	80–100	0.09	0.22**	0.41	0.25	0.55	0.21	0.14*	1.87**	0.62
ZJ										
	0–20	0.19	0.92	0.50**	1.11	0.13*	0.17	0.23	3.25	1.54
	20–40	0.07	0.54	0.34*	1.09	0.15*	0.07	0.25	2.51*	1.53
	40–60	0.11*	0.34	0.51	1.07	0.08**	0.12	0.17*	2.4*	1.45
	60–80	0.13**	0.27	0.26	0.57*	0.05**	0.05	0.22*	1.55**	1.48
	80–100	0.17	0.35	0.42*	0.76*	0.03	0.27**	0.24**	2.24*	1.53

Note: * indicates a significant difference at the 0.05 level (two-sided *t*-test), and ** indicates a significant correlation at the 0.01 level (two-sided *t*-test).

As shown in Fig 4, the pH decreased from an initial range of 8.21–8.35 to 7.71–7.88 in the CG profile. Overall, the pH exhibited a gradual increasing trend from the topsoil to the bottom soil layer. Additionally, the conductivity decreased from an initial range of 0.95–1.14 ms/cm to 0.45–0.68 ms/cm and decreased in each soil layer in the following order from largest decrease to smallest decrease: 80–100 cm, 60–80 cm, 40–60 cm, 0–20 cm and 20–40 cm. The total soluble salt content decreased from an initial range of 2.63–2.81 g/kg to 2.28–2.51 g/kg and exhibited a similar declining trend in each layer of the soil profile except the fourth layer, in which it increased after the third irrigation. The largest reduction in this parameter occurred in the 80–100 cm layer, whereas the smallest reduction occurred in the 20–40 cm layer.

In Fig 5, the pH decreased from an initial range of 8.36–8.54 to 7.73–7.96 in the ZJ profile and decreased in each soil layer in the following order from largest to smallest: 40–60 cm, 20–40 cm, 0–20 cm, 80–100 cm, and 60–80 cm. Compared with the initial pH of the soil, the pH changes in each layer were not obvious over the course of the seven irrigation applications. The conductivity decreased from an initial range of 1.58–1.68 ms/cm to 1.45–1.54 ms/cm and decreased in each soil layer in the following order: 0–20 cm, 20–40 cm, 40–60 cm, 60–80 cm, and 80–100 cm. The total soluble salt content decreased from an initial range of 2.81–4.03 g/kg to 2.56–3.28 g/kg and decreased in each layer of the soil profile in the following order: 0–20 cm, 20–40 cm, 40–60 cm, 60–80 cm, and 80–100 cm.

Overall, compared with those of the CG soil profile, the pH, conductivity, total soluble salt in each layer of the ZJ soil profile were higher because of the high initial values in ZJ.

### Salt ion changes in the soil after irrigation

Based on an analysis of the salt ions in the soil of Huanghua, the main cation is Na^+^, and the main anions are Cl^-^ and SO^4-^. The contents of other ions are relatively low. Based on our analysis, the migration of K^+^ was influenced by moisture migration. SO_4_^2-^ and Ca^2+^, due to their higher charges, were more strongly adsorbed to soil colloids and less affected by the migration of irrigation water. The following analysis focuses on K^+^, Na^+^, and Cl^-^ because the effects of water migration were notable.

**Fig 6:**
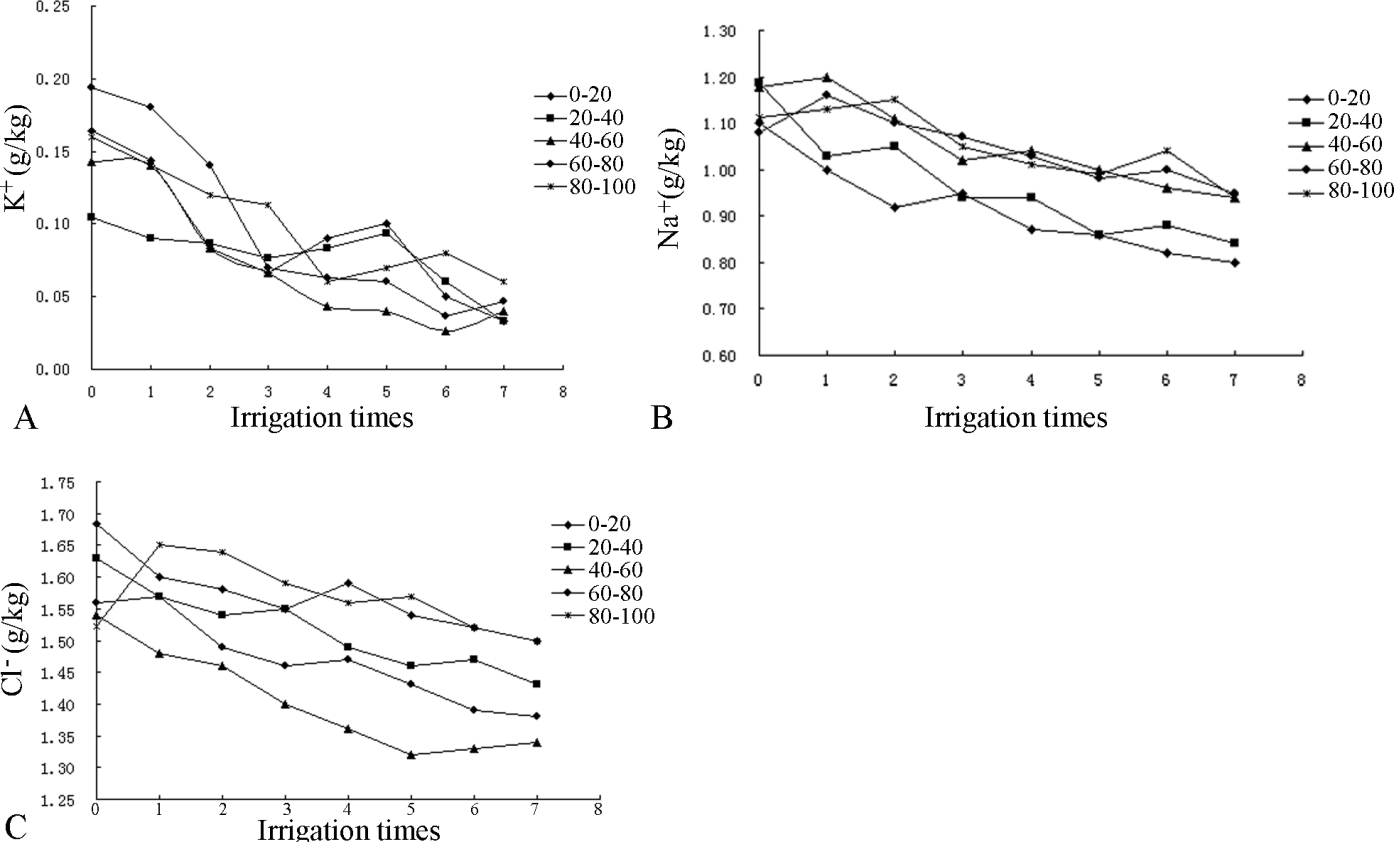
Changes in the salt ion contents of the CG profile.

As shown in Fig 6, K^+^ was concentrated in the 0–20 cm and the 60–80 cm layers of the CG profile. As the number of irrigations increased, the content of K^+^ initially decreased in each soil layer and then gradually decreased in each layer except the second, in which the content remained relatively stable. Notably, the K^+^ content declined quickly after the second and third irrigations and increased slightly after the fourth irrigation due to soil resalinization. Eventually, the K^+^ content began to stabilize.

The initial Na^+^ contents were similar. After the first irrigation, the Na^+^ content decreased in the first two layers and increased in the next three layers, indicating leaching of this ion. However, the leaching did not affect the lower soil layers. As the number of irrigations increased, the Na^+^ content in each layer generally decreased gradually. Notably, the Na^+^ contents in the 20–40 cm, 60–80 cm and 80–100 cm layers increased again after the fifth irrigation due to soil resalinization.

The Cl^-^ content in the soil of the CG profile mainly varied between 1.52 and 1.68 g/kg, with the lowest content in the 80–100 cm layer. After the first irrigation, the Cl^-^ contents in the first four layers all decreased, whereas the content in the fifth layer increased, indicating leaching into the lower soil layers. After the fourth irrigation, soil resalinization was observed in the 0–20 cm and 80–100 cm layers. After the final irrigation, the Cl^-^ contents generally decreased.

**Fig 7:**
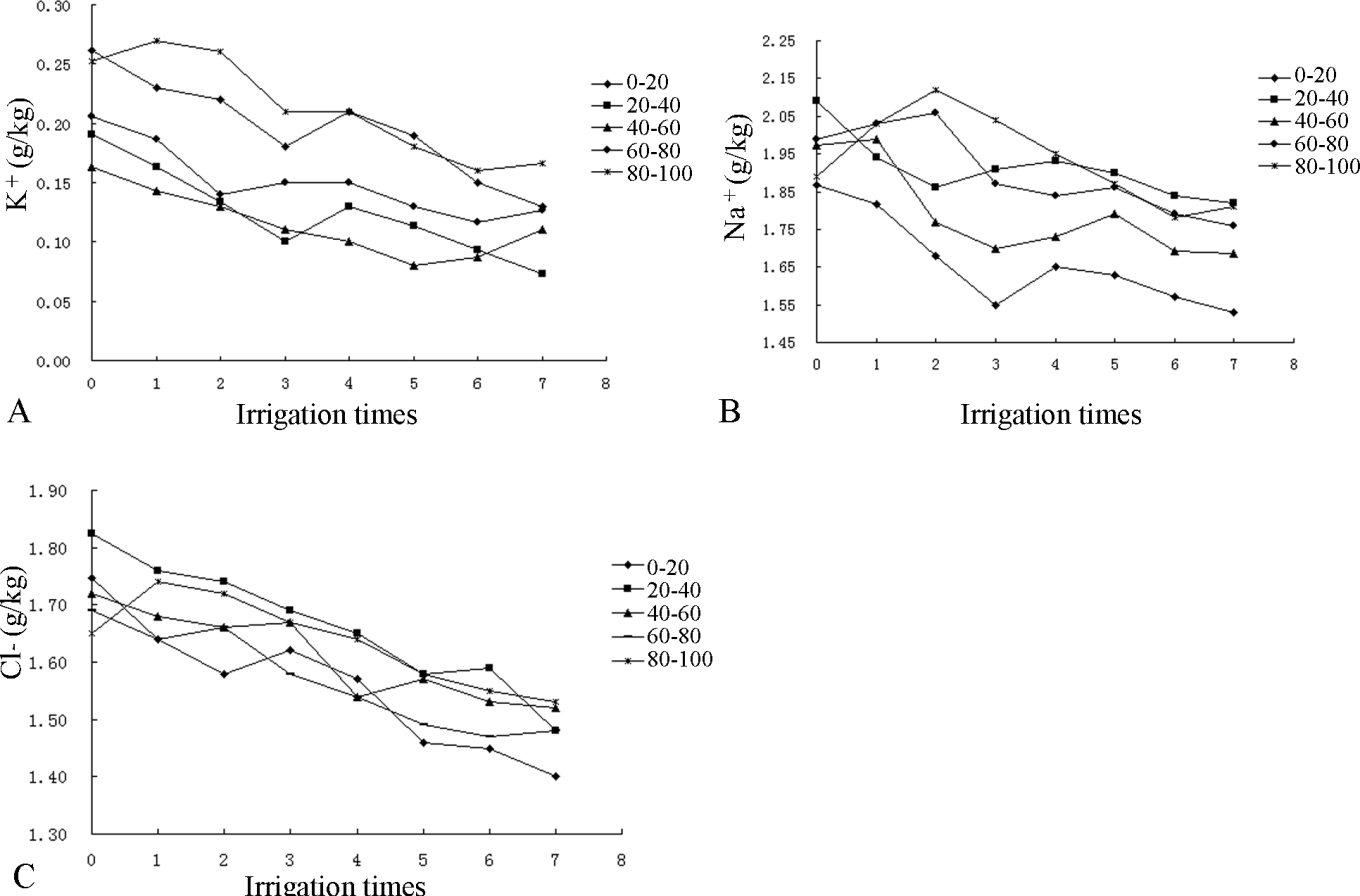
Changes in the salt ion contents of the ZJ profile.

As shown in Fig 7, the content of K ^+^ in the ZJ profile mainly varied between 0.16 and 0.25 g/kg. After the first irrigation, the K ^+^ contents in most layers increased, with the exception of the fifth layer, in which it decreased. After the fourth irrigation, soil resalinization was observed, especially in the topsoil. After the seventh irrigation, the K^+^ content again decreased. Overall, the K^+^ content in the soil exhibited a decreasing trend, with the highest content in the topsoil.

The content of Na^+^ in the ZJ profile varied between 1.85 and 2.10 g/kg, and Na^+^ was concentrated in the 20–40 cm, 40–60 cm, and 80–100 cm layers. After the first irrigation, the Na^+^ contents in the first three layers all decreased, while the contents in the 60–80 cm and 80–100 cm layers increased, especially in the 80–100 cm layer. This observation suggests that Na^+^ was not leached beyond the 100 cm soil layer. After the third irrigation, soil resalinization was observed, especially in the 0–20 cm layer. Overall, the Na^+^ content in the soil exhibited a decreasing trend, with the lowest content in the topsoil.

The content of Cl^-^ in the ZJ profile mainly varied between 1.65 and 1.81 g/kg, with the highest content in the 20–40 cm layer. After the first irrigation, the Cl^-^ content increased in the 80–100 cm layer but decreased in the other layers. Thus, Cl^-^ was leached to the bottom soil layers, thereby increasing the Cl^-^ content in the fifth layer. After the third irrigation, soil resalinization was observed. With continued irrigation, Cl^-^ exhibited a decreasing trend in all layers, with the lowest content in the topsoil.

These indoor experiments simulating the dynamic changes in water and salinity in saline-alkali soil revealed that after multiple irrigations of the soil column, the pH, conductivity, and total soluble salt content of the percolation samples were all higher than those of the initial water samples. With additional irrigation applications, these parameters gradually decreased in the percolation samples, consistent with the results in [23].

After seven rounds of irrigation, samples of the various soil layers were compared with the initial soil samples. The pH, conductivity, and total soluble salt content values decreased overall, reflecting desalination in the simulation. When the 20–70 cm layer of the soil column was irrigated, the total salt content in the bottom layer of the soil column became 2.21–3.42 mg/L, representing a general decrease from initial values of 2.35–3.84 mg/L [23]. With increasing irrigation applications, the contents of all salt ions in the soil from Huanghua increased initially then decreased, reflecting resalinization and the considerable impact of soil moisture transport on K^+^, Na^+^, and Cl^-^. These lower contents might have been caused by the decrease in salinity associated with water loss during repeated irrigation. Eventually, salt ions migrated upward due to evaporation, resulting in resalinization. However, the amount of salt movement due to resalinization was smaller than that migrating downward, and the net result was a decrease in the salt contents. Li et al. found that the Ca^+^ content was coupled with the variations in water and temperature in soil and that the transport of K^+^ and SO_4_^2-^ in soil was greatly impacted by water movement [24]. Furthermore, Li et al. found that under the same drainage conditions in a cotton field with variable salinity contents, increased irrigation might not necessarily lead to greater desalination [25]. In this study, the irrigation amount was determined according to the local precipitation and water demand of crops, indicating that the desalination in the ZJ and CG profiles is representative. The results of the current study will serve as a reference for agricultural production in areas with similar soil conditions.

## Conclusions

Indoor simulation experiments of soil water and salinity dynamics after irrigation demonstrated that irrigation-induced leaching decreased the salinity in the soil layers and that the content of ions that could be transported decreased with repeated irrigation. The soil conductivity, pH and total soluble salt content in each soil layer decreased slightly, and these parameters were higher in each layer of the ZJ profile than in the corresponding layer of the CG profile.

The water content in the soil did not change significantly with additional irrigation and exhibited no clear pattern of variation in each layer after each irrigation. The variations in the total soluble salt contents in the ZJ and CG profiles were similar, i.e., an initial increase followed by a decrease. The total soluble salt content in the CG profile was higher than that in the ZJ profile.

This study simulated desalination under certain irrigation conditions, and the salts in each layer of the soil column constantly migrated downward with water due to leaching. As a result, the topsoil became desalinated. In addition, we simulated the dynamic changes in the water and salinity in the soil, thereby providing technical guidance for the comprehensive management of saline-alkali soils under the local irrigation conditions. Future investigations will utilize outdoor experiments to explore the dynamic changes in the water and salinity in soil profiles from the study area to determine rational irrigation schemes and provide a theoretical basis for field irrigation and management of saline-alkali soils.
